# Evolution of prehistoric dryland agriculture in the arid and semi-arid transition zone in northern China

**DOI:** 10.1371/journal.pone.0198750

**Published:** 2018-08-03

**Authors:** Yige Bao, Xinying Zhou, Hanbin Liu, Songmei Hu, Keliang Zhao, Pia Atahan, John Dodson, Xiaoqiang Li

**Affiliations:** 1 State Key Laboratory of Vertebrate Evolution and Human Origin, Institute of Vertebrate Paleontology and Paleoanthropology, Chinese Academy of Sciences, Beijing, China; 2 Graduate University of Chinese Academy of Sciences, Beijing, China; 3 Shaanxi Provincial Institute of Archaeology, Xi’an, China; 4 Institute for Environmental Research, Australian Nuclear Science and Technology Organisation, Kirrawee DC, New South Wales, Australia; 5 School of Earth and Environmental Sciences, University of Wollongong, Wollongong, New South Wales, Australia; 6 State Key Laboratory of Loess and Quaternary Geology, Institute of Earth Environment, Chinese Academy of Sciences, Xi’an, Shaanxi, China; New York State Museum, UNITED STATES

## Abstract

Based on chronological and archaeobotanical studies of 15 Neolithic and Bronze Age sites from the northern Chinese Loess Plateau and southern Inner Mongolia—the agro-pastoral zone of China–we document changes in the agricultural system over time. The results show that wheat and rice were not the major crops of the ancient agricultural systems in these areas, since their remains are rarely recovered, and that millet cultivation was dominant. Millet agriculture increased substantially from 3000 BC–2000 BC, and foxtail millet evidently comprised a high proportion of the cultivated crop plants during this period. In addition, as the human population increased from the Yangshao to the Longshan periods, the length and width of common millet seeds increased by 20–30%. This demonstrates the co-evolution of both plants and the human population in the region. Overall, our results reveal a complex agricultural-gardening system based on the cultivation of common millet, foxtail millet, soybeans and fruit trees, indicating a high food diversity and selectivity of the human population.

## Introduction

The development of agriculture is fundamental to the large-scale transition of human societies from a hunting and gathering to a settled lifestyle, in both the Old and New World [1–4]. Settled communities permitted humans to observe and experiment with plants and to understand how they grew, which greatly reduced food shortages and directly resulted in both population increases [5–8] and the co-evolution of both human society and crop production [9]. In recent years, the increasing amount of research on prehistoric agriculture has significantly improved our understanding of the origins of agriculture in the Old World [7–18]. Specifically, ancient agriculture has emerged as a major area of interest in Asian archaeology [19]. Chinese agricultural traditions have two independent areas of origin: rice agriculture in the lower Changjiang River Basin, and dryland agriculture in the Yellow River Basin [20–23]. Dryland agriculture was dominated by two principal crops, foxtail millet (*Setaria italica*) and common millet (*Panicum miliaceum*), which were both domesticated probably as early as 8000 BC in North China [7,20,21]. The spread of these two crop plants into Western Asia and eventually Europe occurred mainly from 2000 BC-1000 BC, when wheat was widely planted in northern China (no later than 2100 BC); in addition, common millet spread to Central Asia along the Silk Road at about the same time [24]. The cultivation of both plants directly reflects increasing socioeconomic development and technology proficiency [7]. The main advantages of foxtail and common millet, which are C_4_ plants, is that they have high drought tolerance and can be grown in nutrient-poor soils. In most cases, rain-fed millet produces good harvests under conditions of low summer rainfall and it can grow in almost any soil type [7]. Nevertheless, foxtail and common millet cultivation first developed in the North China Plain, where rainfall was plentiful, during the early and middle Holocene. However, in the context of research into crop domestication and evolution [25–30], how millet agriculture first expanded from its center of origin to the adjacent dryland area and became adapted to an arid environment is poorly known.

The agro-pastoral zone in North China marks the border between traditional agricultural societies and nomadic societies, which has shifted backwards and forwards between agricultural and nomadic nations during the last several thousand years. It is a key area for studying the early civilization of ancient China. In the middle Neolithic, farmers of the Yangshao culture moved from the humid and sub-humid monsoon area of central China to occupy the agro-pastoral zone, and the local agricultural society became extremely prosperous during the late Neolithic and early Bronze Age transition [31–33]. In addition to possible climatic factors, the prosperity of the ancient agricultural societies of the arid agro-pastoral zone may have been due to innovations in the local agricultural system. These innovations probably facilitated the expansion of millet cultivars across the steppe and deserts of Eurasia, which eventually provided the agricultural basis of the nomadic pastoralists from Asia to Eurasia, and resulted in their becoming global crops by around 2000 BC [7,24,34]. However, the Neolithic and Bronze age agricultural systems in the agro-pastoral zone of China are still poorly documented.

**Fig 1:**
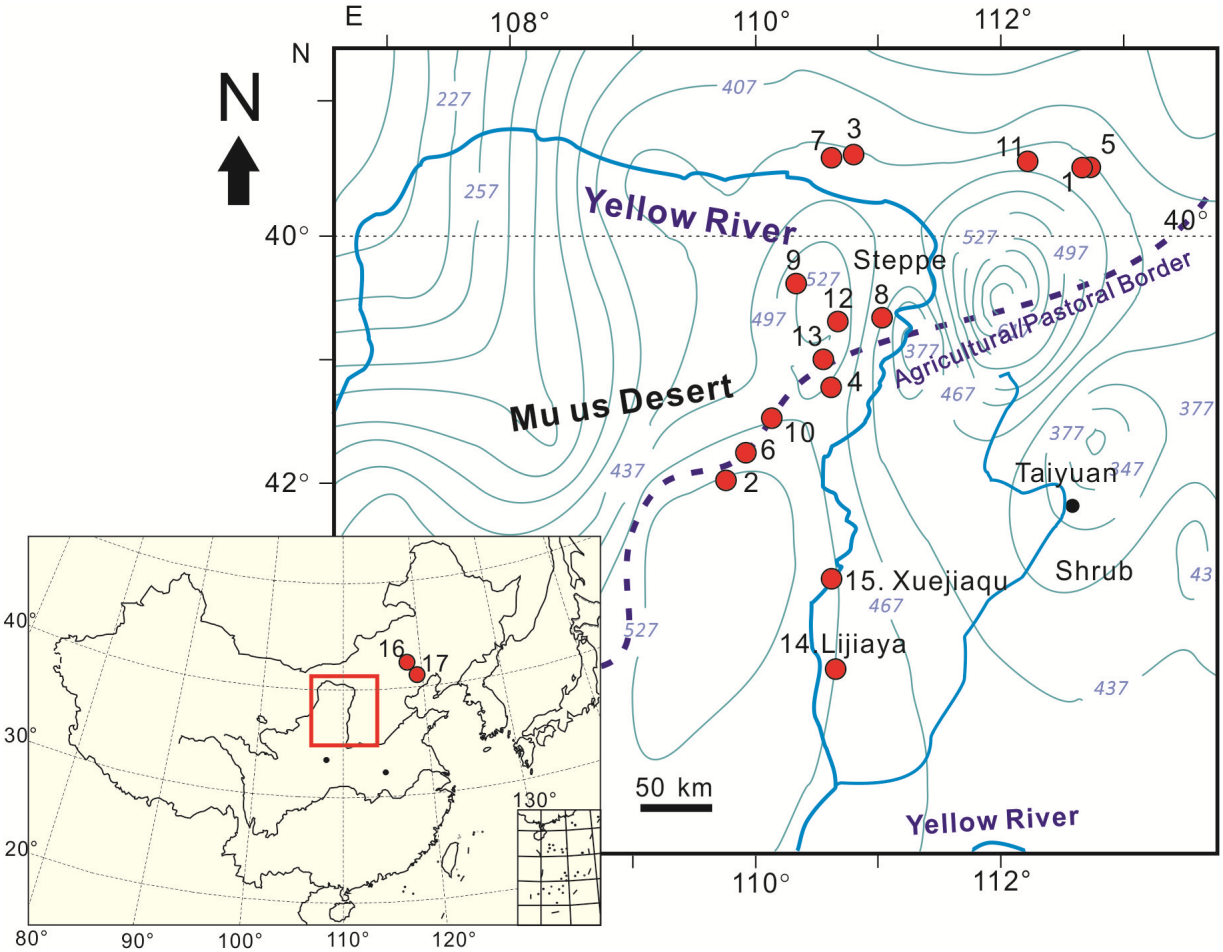
Site locations and modern precipitation in the study area, the current agricultural-pastoral border of China. 1. Shihushan, 2. Xiaojiamao, 3. Xiyuan, 4. Zhaimao, 5. Wangmushan (overlapping with Shihushan), 6. Huoshiliang, 7. Ashan, 8. Dakou, 9. Zhukaigou, 10. Xinhua, 11. Laohushan, 12. Erliban, 13. Shimao, 14. Lijiaya, 15. Xuejiaqu, 16. Xiajiadian, 17. Chengzishan. The precipitation contours are cited from China Meteorological Data Service Center, using the data for 2017 (http://data.cma.cn/site/index.html).

Here, we present the results of geochronological and archaeobotanical studies of 15 Neolithic and Bronze Age sites from the northern Chinese Loess Plateau (CLP) and southern Inner Mongolia (Fig 1). Our principal aim was to shed light on the pattern of agricultural change in the drylands of the East Asian temperate zone, using quantitative analysis of seeds and a study of seed morphology of flotation samples from Neolithic and Bronze Age sites in the region. In addition, we integrate our findings with published archaeobotanical data from the agro-pastoral zone to the east to provide a comprehensive picture of the process of agricultural development from 4000 BC-1000 BC in the agro-pastoral zone of China.

## Background setting and study sites

The central agro-pastoral zone of China mainly comprises the southern part of Inner Mongolia and the northern part of the CLP and is an important pathway between northeast and northwest China. The central agro-pastoral zone has a continental monsoon climate, with mean annual temperature of 7 to 12 °C and mean annual precipitation from 300 to 450 mm. The modern vegetation is mainly *Artemisia-Bothriochloa* grassland and shrubland, with *Zizyphus*, *Vitex*, *Rosa* and *Hippophae*, and woodland consisting of *Platycladus* and *Pinus* [35].

The regional rainfall is relatively low and was sensitive to past climatic oscillations [36–38]. Paleoclimatic studies indicate a higher precipitation and high lake levels in the area during the Neolithic and Bronze Ages, caused by strong summer monsoon circulation during the early and middle Holocene [39]. However, the vegetation distribution during the middle Holocene was still dominated by shrub-grassland and steppe, with sparse woodland near riverbeds, which may reflect the high temperatures and high evaporation [39].

In recent decades, thousands of sites belonging to a series of Neolithic and Bronze Age cultures dated to 6000 BC-1500 BC have been discovered in the region. In sequence, the cultures are as follows: The first period of Hougang culture, Shihushan type; Yangshao culture, Lower Wangmushan type; Haishengbulang culture; Xinhua culture; Ashan culture; Laohushan culture; Dakou culture; Zhaimao culture; Zhukaigou culture; Shimao culture; Guifang culture (Table 1). Most of the sites belong to the Late Longshan or Zhukaigou cultures (2500 BC-1800 BC) [40,41]. Shimao (2036 BC-1519 BC) is the best known and most extensive site in the region, with an area of over 4.25 km^2^. It is surrounded by three stone walls that were built for defense against invasion [39] and is the largest prehistoric walled city found in China. In terms of social structure and residential characteristics, Shimao consisted of several settlement clusters; by 2000 BC it was highly organized with a well-developed arid-land agricultural-pastoral economy [40]. Other sites, such as Zhukaigou (2147–1190 BC), Dakou (2342–1903 BC), Xuejiaqu (1449–1031 BC), Ashan (2582–2121 BC) and Zhaimao (2135–1939 BC), were subsequently discovered[42–44]. Agricultural development and technological exchange within the northern steppe region are reflected by the consistency of the composition and abundance of agricultural remains at these sites, including the carbonized remains of crop plants and grains, and farm implements [45–47].

**Table 1:** Study sites and their ^14^C ages.

Site/Sample code	Lab code		Location	Culture	Material	Calendar age uncalibrated yrs BC	1s error	Calibrated age (most probable) cal BC/2 sigma	County	Reference	Laboratory
**Shihushan**	SI3001	OZP087	40°30′N;112°43′E	The first period of Hougang culture, Shihushan type	Sheep	3740	40	4696–4491	Liangcheng	This study	ANSTO
	SI2998	OZP086			Sheep	3695	35	4617–4454			
	SI2997	OZP085			Sheep	3665	40	4604–4441			
**Xiaojiamao**	-	-	38°8′N;109°20′E	Unknown	Charcoal	-	-	3500–3000	Xiaojiamao	Liu, 2011	ANSTO
**Wangmushan**	-	95WST14H6	40°30′N;112°43′E	Yangshao culture, Lower Wangmushan type	Charcoal	2520	70	3376–3010	Liangcheng	State Cultural Relics Bureau, 1986	ANSTO
**Xiyuan**	Xiyuan-1	OZP042	40°35′N;110°17′E	Haishengbulang culture	Common millet	2285	50	3028–2858	Shaerqin	This study	ANSTO
**Xinhua**	-	-	38°38′N;110°2′E	Xinhua culture	-	2030	120	2890–2278	Shenmu	Shaanxi Provincial Institute of Archaeology,1999	ANSTO
**Ashan**	SI2894	OZP077	40°35′N;110°13′E	Ashan culture	Pig	2015	30	2582–2470	Baotou	This study	ANSTO
	SI2987	OZP078			Cattle	1975	30	2577–2454			
	SI2989	OZP079			Dog	1960	30	2504–2399			
	Ashan-2	OZP043			Common millet	1770	35	2295–2121			
**Laohushan**	-	WB84~44	40°30′N;112°16′E	Laohushan culture	Charcoal	1870	70	2493–2141	Liangcheng	State Cultural Relics Bureau, 1986	ANSTO
**Erliban**	-	ZK~2241	39°27′N;110°50′E	Xiongnu culture	Charcoal	1810	65	2466–2124	Jungar Banner	Chinese Archaeology,1988	ANSTO
**Dakou**	SI2940	OZO945	39°25′N;111°8′E	Dakou culture	Human	1780	40	2342–2121	Jungar Banner	This study	ANSTO
	SI2942	OZO946			Human	1720	45	2231–2010			
	SI2943	OZP083			Pig	1635	35	2058–1903			
**Zhaimao**	JM-2012-CM	Beta462799	38°51′N;110°36′E	Zhaimao culture	Common millet	1650	30	2135–1939		This study	BETA
**Huoshiliang**	SI2890	OZP084	37°22′N;108°13′E	Unknown	Horse	2145	40	2878–2619	Yulin	This study	ANSTO
	SI2900	OZO952			Pig	1605	45	2060–1877			
	SI2891	OZO951			Ovis	1570	50	2036–1755			
	SI2908	OZO950			Bone	1555	40	1983–1768			
**Zhukaigou**	ZKG4	OZM232	39°39′N;110°26′E	Zhukaigou culture	Charcoal	1680	40	2147–1950	Linta	This study	ANSTO
	ZKG1	OZQ354			Fruit	1610	35	2041–1885			
	ZKG-S-29	OZQ360			Charcoal	1560	40	2023–1860			
	ZKG2	OZQ355			Millet seed	1545	35	1976–1766			
	SI1573	OZN201			Bone	1505	30	1912–1746			
	ZKG human 2	OZM221			Human	1500	-	1854–1772			
	SI1575	OZN202			Bone	1015	30	1316–1190			
**Shimao**	SI2936	OZO954	39°4′N;110°28′E	Shimao culture	Human	1570	50	2036–1755	Shenmu	This study	ANSTO
	SI2938	OZO956			Human	1555	45	2023–1761			
	SI2937	OZO955			Human	1540	45	1981–1746			
	SI2935	OZO953			Human	1515	50	1972–1734			
	Shimao-2	OZO041			Common millet	1280	-	1611–1519			
**Chengzishan**	-	OZK414	41°14′N;119°32′E	Lower Xiajiadian culture	Charcoal	1400	45	1783–1610	Lingyuan	Zhao, 2009	ANSTO
		OZK411			Charcoal	1360	70	1783–1499			
		OZK413			Charcoal	1340	60	1771–1496			
		OZK410			Charcoal	1335	50	1701–1504			
**Xuejiaqu**	-	ZK~1184	37°33′N;110°29′E	Unknown	Charcoal	1030	80	1449–1031	Suide	Xu, 1988	ANSTO
**Lijiaya**	Lijiaya-1	OZP044	37°5′N;110°27′E	Guifang culture	Foxtail millet	930	40	1233–1011	Gaojie	This study	ANSTO
**Xiajiadian**	-	OZK430	42°20′N;118°58′E	Upper Xiajiadian culture	Charcoal	560	50	816–539	Chifeng	Zhao, 2009	ANSTO
		OZK427			Charcoal	525	45	801–512			
		OZK428			Charcoal	495	45	792–477			
		OZK429			Charcoal	490	45	789–475			
		OZK426			Charcoal	435	50	671–405			

The ages were calibrated used Calib 510, and we selected the most probable calibrated age.

The locations of the 15 study sites (109°20′~112°43′E, 37°5′~40°35′N), together with Chengzishan (1783–1504 BC) and Xiajiadian (816–405 BC) [48], in the western Liaoning area, are shown in Fig 1 and listed in Table 1. Most of the sites are in riparian locations in the steppe and shrub grassland zone in southern Inner Mongolia and northern Shaanxi Province.

## Methods

**Fig 2:**
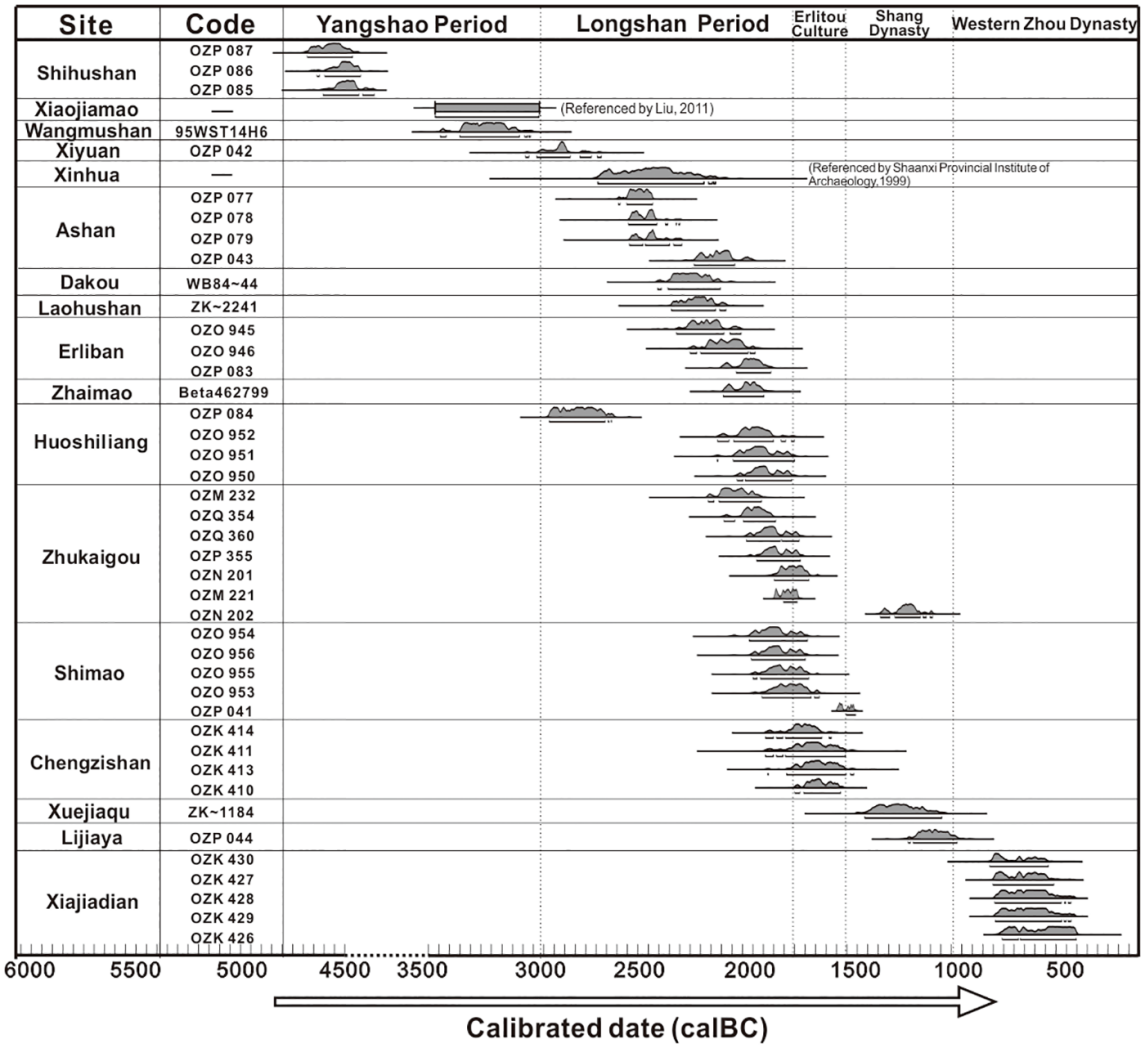
Results of AMS radiocarbon dating of samples from cultural horizons at the studied archaeological sites. All results are reported with 2 sigma error. ^14^C ages are calibrated to calendar years using Calib Rev 5.1 and OxCal v4.3.6, with the r:5 IntCal 13 atmospheric curve.

From among the 15 sites in the Chinese Loess Plateau area, 52 soil samples were collected from sections containing cultural horizons that were deposited within stratigraphic layers or from ancient ash pits which are filled with gray soil and contained numerous plant remains and pottery fragments [49–52]. The volume of each sample was 40 L in the cultural layers and 20 L in the ash pits. The cell number per square inch of flotation sieve was 50 (0.3 mm mesh). Analysis of the coarse fraction revealed only coarse rock fragments, animal bones and occasional pottery shards. The floated samples were separated and identified under a stereoscopic microscope in the laboratory of the Institute of Vertebrate Paleontology and Paleoanthropology, CAS, Beijing (Fig 2). In addition, we conducted morphological studies based on measurements of the length and width of the seeds under the microscope (Table 2). A total of 268 foxtail millet seeds and 123 common millet seeds from six sites (Xiaojiamao (3500–3000 BC), Xinhua (2890–2278 BC), Dakou, Zhaimao, Zhukaigou, Shimao) were selected for the morphological studies. At least five individual seeds of each millet type were measured from each individual site.

**Table 2:** Morphological characteristics (average length and width) of carbonized seeds of foxtail millet (*Setaria italica*) and common millet (*Panicum miliaceum*).

Site and crop Species		Xiaojiamao	Xinhua	Dakou	Zhaimao	Zhukaigou	Shimao
***Setaria italica***	Length (Average/mm)	1.492	1.403	1.403	1.242	1.438	1.299
	Width (Average/mm)	1.341	1.237	1.236	1.115	1.277	1.124
	Length/Width (mm)	1.112	1.144	1.135	1.119	1.131	1.155
***Panicum miliaceum***	Length (Average/mm)	1.582	1.656	1.689	1.719	1.828	1.785
	Width (Average/mm)	1.379	1.399	1.588	1.701	1.653	1.589
	Length/Width (mm)	1.147	1.183	1.072	1.010	1.111	1.123

We selected whole, intact seeds for measurement. For foxtail millet, we analyzed the following (site and number of seeds in parentheses): Xiaojiamao (27), Xinhua (18), Dakou (23), Zhaimao (80), Zhukaigou (84), Shimao (36); and for common millet: Xiaojiamao (14), Xinhua (11), Dakou (10), Zhaimao (13), Zhukaigou (54), Shimao (21).

Samples from the 15 sites were selected for AMS ^14^C dating (Table 1). The samples included 3 animal bones from Shihushan (4696 BC-4441 BC), 1 foxtail millet seed from Xiyuan (3028 BC-2858 BC), 4 animal bones from Ashan, 3 animal and human bones from Dakou, 1 common millet seed from Zhaimao, 4 animal bones from Huoshiliang (2878–1768 BC), 7 samples of charcoal, foxtail millet and human bones from Zhukaigou, 4 human bones and 1 common millet seed from Shimao, and 1 foxtail millet seed from Lijiaya (1233 BC-1011 BC) (Table 1).

The radiocarbon ages were measured by accelerator mass spectrometry in the laboratory of the Australian Nuclear Science and Technology Organization (ANSTO) in Sydney, Australia, and by Beta Analytic in Miami, USA. The samples were pretreated by washing in acid/alkali solutions and rinsing to neutral pH.

## Results

The radiocarbon dating results show that the oldest site is Shihushan and the youngest is Lijiaya. Only three sites (Shihushan, Xiaojiamao, Wangmushan) are of Yangshao age, which is usually dated to 5000 BC-3000 BC. Most of the AMS ^14^C ages are from 2500 BC-1500 BC, corresponding to a prosperous period of dryland agricultural societies in the north Chinese Loess Plateau. Xuejiaqu and Lijiaya are dated to 1500 BC-1000 BC, which is contemporaneous with the Shang Dynasty.

From the 52 flotation samples taken from the 15 study sites, we selected and identified 3110 charred grains, belonging to 14 genera or species, (Table 3). By comparison with sites from traditional farming areas, seeds are scarce in the cultural layers of sites in the steppe region. No seeds or fruit stones were recovered from Wangmushan and Xiyuan, and no crops seeds were recovered from Shihushan, Erliban and Lijiaya.

**Table 3:** Counts of crop seeds, weed seeds, kernels and nut shells in the studied samples.

Type & Site	Crops									Shells				
	*Setaria italica*	*Panicum miliaceum*	*Glycine soja*	*Viola*	*Medicago Linn*.	Poaceae	Amaranthaceae	*Lespedeza*	*Hibiscus trionum L*.	*Castanea*	*Corylus*	*Pinus Linn*	*Prunus persica*	*Prunus armeniaca*
**Shihushan**	0	0	0	0	0	0	0	0	0	0	1	0	0	1
**Xiaojiamao**	101	201	0	0	0	0	5	5	0	0	0	0	0	16
**Wangmushan**	0	0	0	0	0	4	0	1	0	0	0	0	0	0
**Xiyuan**	0	0	0	0	0	0	0	0	0	0	0	0	0	0
**Xinhua**	28	5	5	0	0	1	36	0	0	0	0	0	0	10
**Ashan**	14	10	0	0	0	0	0	0	0	0	0	0	0	2
**Laohushan**	2	0	0	0	0	4	0	1	0	0	0	0	0	0
**Erliban**	0	0	0	0	0	0	15	0	12	0	0	0	0	2
**Dakou**	66	65	0	0	0	0	1	0	0	0	0	0	0	0
**Zhaimao**	615	99	0	1	0	0	0	0	1	5	0	0	0	13
**Huoshiliang**	23	10	0	0	0	0	0	0	0	0	1	1	0	26
**Zhukaigou**	389	335	29	0	0	0	5	0	0	5	0	0	4	9
**Shimao**	173	15	11	1	1	4	0	0	0	0	1	0	0	2
**Chengzishan**	314	98	0	0	0	0	0	0	0	0	0	0	0	0
**Xuejiaqu**	2	2	0	0	0	0	0	0	0	0	0	0	0	0
**Lijiaya**	0	0	0	0	0	0	0	1	0	0	1	0	0	3
**Xiajiadian**	151	157	0	0	0	0	0	0	0	0	0	0	0	0
**Total**	**1878**	**997**	**45**	**2**	**1**	**13**	**62**	**8**	**13**	**10**	**4**	**1**	**4**	**84**

The 3110 cultivated plant seeds consist primarily of three species: foxtail millet (*Setaria italica*), common millet (*Panicum miliaceum*) and soybean (*Glycine soja*). Foxtail millet and common millet account for the highest proportion, with foxtail millet comprising 60.39% and common millet 32.06% of the total (Table 2). The percentage of foxtail millet seeds increased from 30.79% in the Yangshao Period (Xiaojiamao) to 50–83.79% in the late Longshan Period (Dakou, Shimao, Zhaimao), indicating a trend of increasing importance of millet cultivation in the agricultural system.

**Fig 3:**
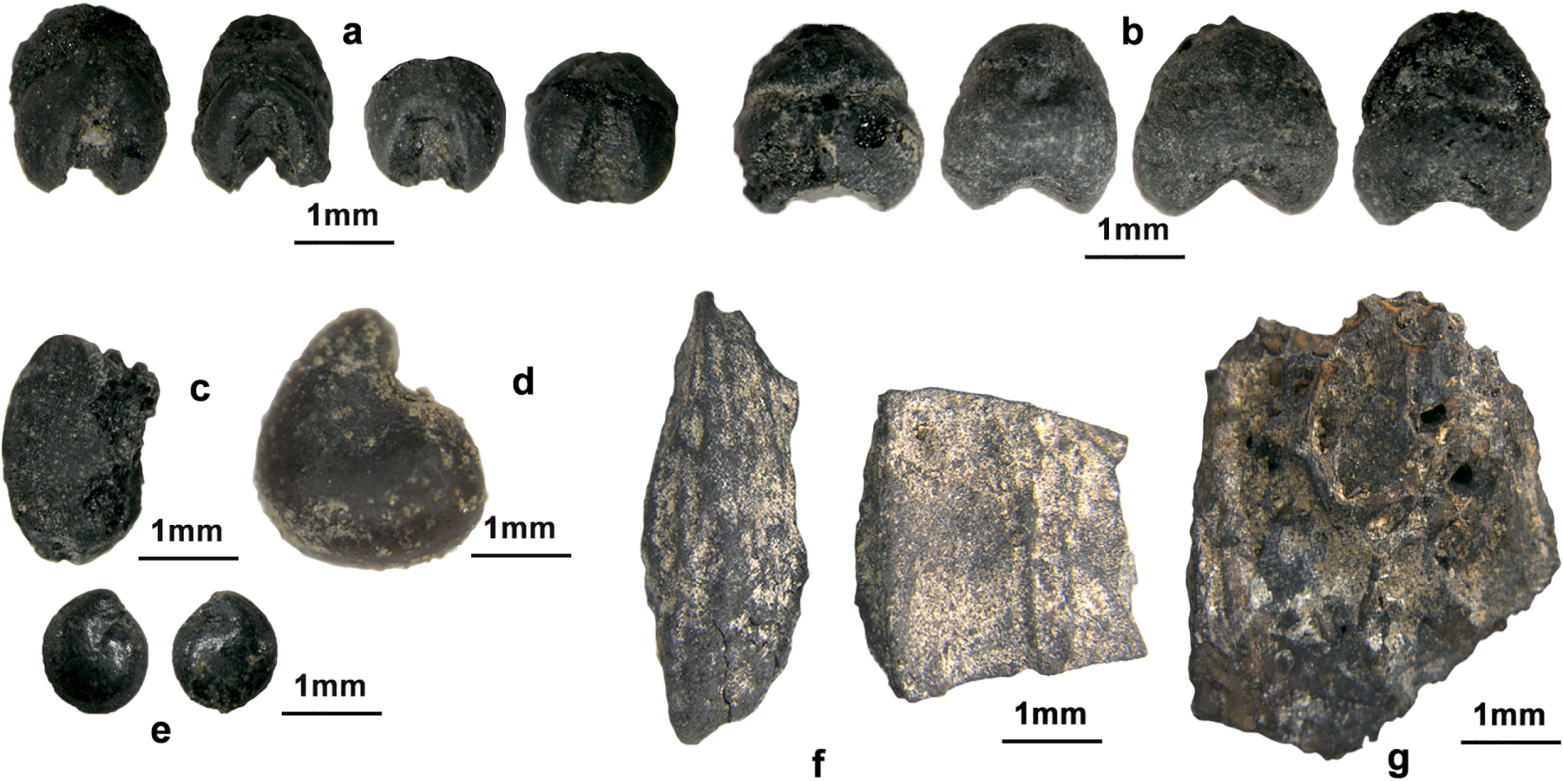
Stereoscan photographs of seeds and fruit stones. a. *Setaria italica*, Zhukaigou; b *Panicum miliaceum*, Zhukaigou; c. *Glycine soja*, Zhukaigou; d. *Hibiscus trionum*, Erliban; e. *Chenopodium*, Xinhua; f. *Prunus armeniaca*, Shimao; g. *Prunus persica*, Zhukaigou. a and b are cereal crops, c-e are the seeds of wild plants, and f and g are probably from cultivated trees.

**Fig 4:**
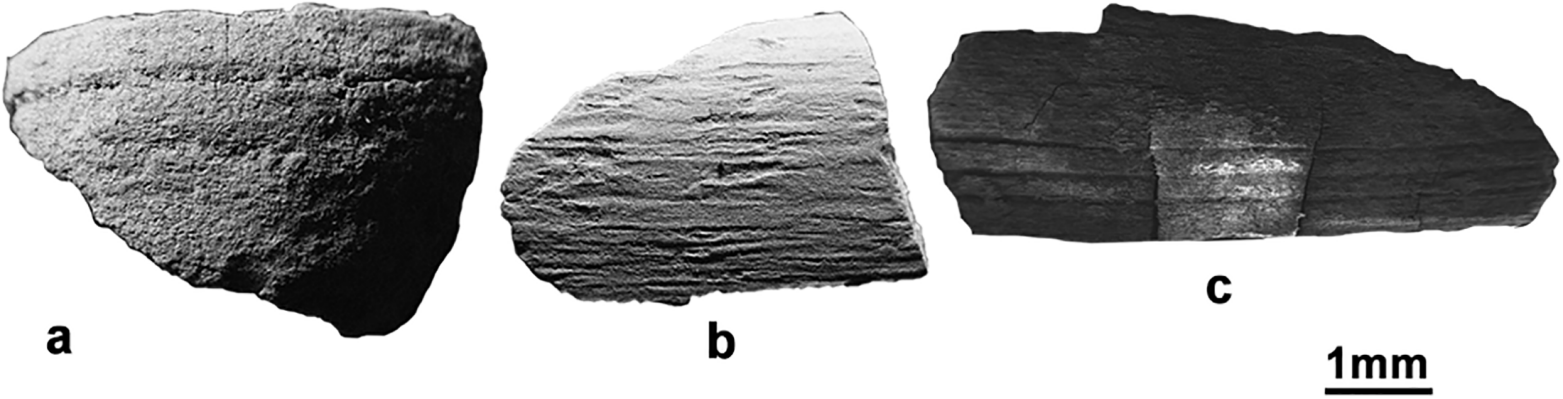
Stereoscan photographs of nut shells (suspected). These suspected fruit shells have radians and ornamentation on the surface. The shell remains were too fragile to be identified accurately; however, we could identify three relatively intact specimens: a. *Corylus*, Huoshiliang; b. *Pinus*, Huoshiliang; c. *Castanea*, Zhaimao.

Several seeds of *Glycine soja* were found at Shimao, Zhukaigou and Xinhua. In addition, several seeds of typical weed species of *Fabaceae*, including *Melilotus*, *Lespedeza* and *Medicago*, occurred sporadically in the samples. A high proportion of *Chenopodium* was present at the sites; however, whether *Chenopodium* grew as a weed or was derived from human or animal excreta could not be determined. In addition, seeds, several nut shells and drupe pits, including *Castanea*, *Prunus persica*, *Corylus*, *Prunus armeniaca* and *Pinus*, were found in the samples. *Prunus armeniaca* occurred at the highest frequency and was found at nine sites (Figs 3 and 4).

**Fig 5:**
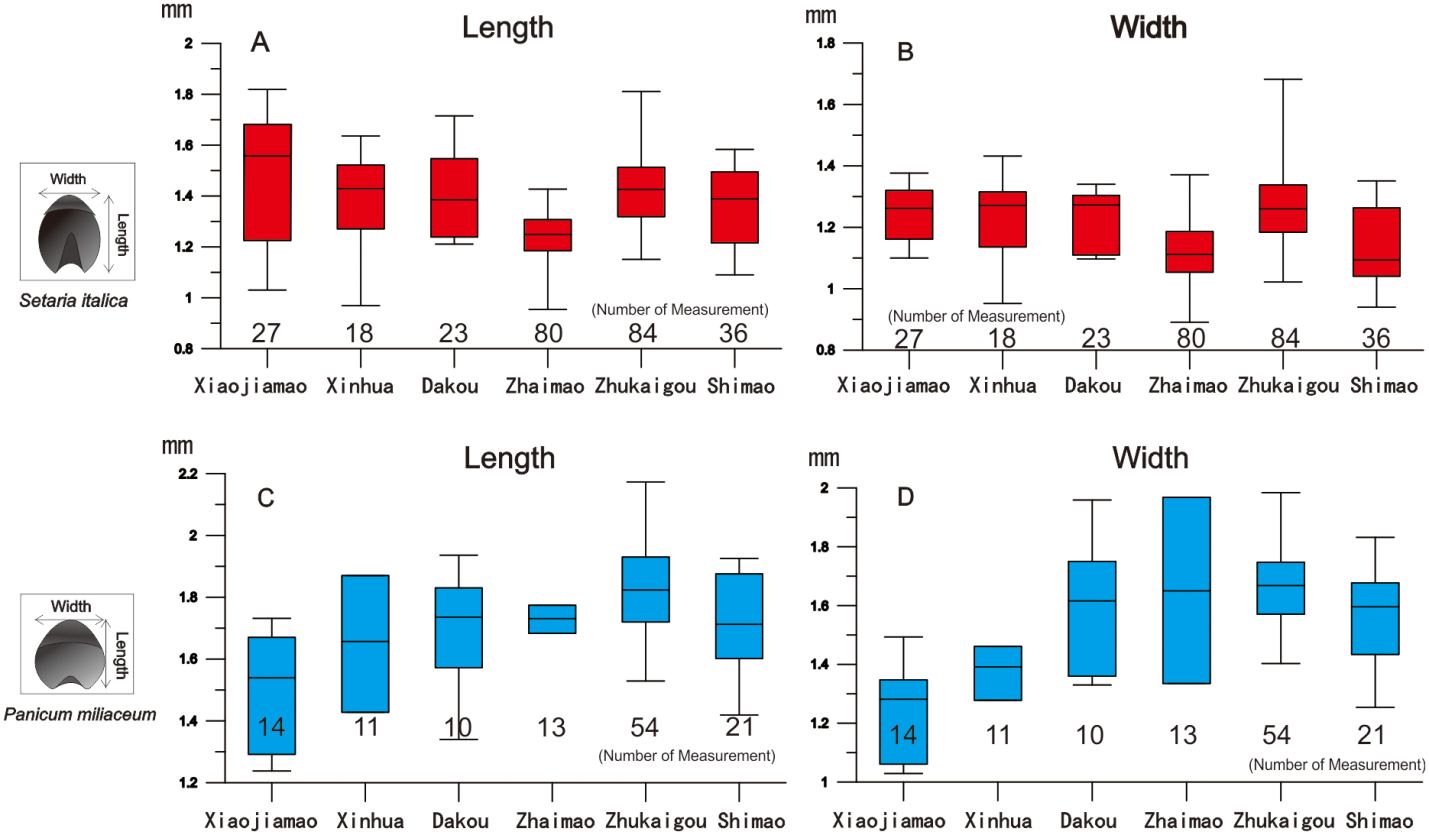
Box plots illustrating changes in the length and width of the seeds of foxtail millet (top) and common millet (bottom) from Neolithic and Bronze Age sites in the agro-pastoral zone of China.

Statistical analysis of the length and width of the foxtail and common millet seeds from six sites revealed an increasing trend from the early to the late period (Fig 5). From Xiaojiamao to Zhukaigou, the length and width of common millet seeds increased significantly, by about 20% and 35%, respectively. However, the increase was less significant in the case of foxtail millet.

## Discussion

Foxtail millet and common millet were the principal crops in the early stage of the development of dryland agriculture in the area; they were first domesticated in the middle and lower reaches of the Yellow River, within the East Asian monsoon region, at about 6000 BC [38]. The average annual rainfall of the area is estimated to have been as high as 800–1000 mm in the early and middle Holocene [53]. During subsequent millennia, millet agriculture expanded stepwise to the marginal regions of the monsoon area where rainfall is lower and the climate less stable. In addition, rice was introduced from the lower Changjiang River basin, and was cultivated in the southern and western parts of the Loess Plateau, adjacent to the present study area, before 3000 BC [54]. Wheat was introduced to China at around 2000 BC from Western and Central Asia and during the next several hundred years its cultivation resulted in a rapid agricultural transformation in the Hexi Corridor [55–57]. At the same time, millet agriculture continued to flourish in the arid and semi-arid zone, near the Hexi Corridor [7,37]. Thus, there is a consistent picture of a broadening agricultural system, with millet, rice, wheat and barley, in the sites of late Neolithic to Bronze Age in central China [54,57].

Our results reveal that the agricultural system was continuously dominated by foxtail and common millet cultivation from the early Yangshao period (5000 BC-3000 BC) to the Bronze Age, and no wheat or rice grains were found at any of the 15 sites. This suggests that the inhabitants developed a cereal-based agricultural system that was adapted to a relatively arid climate. This inference agrees with conclusions based on δ^13^C analyses of human bones from Zhukaigou, Dakou and Xinhua, which show that C_4_-type plants were the main food source within the region [58]. This somewhat narrowly focused system in the agro-pastoral zone during the Neolithic and Bronze Age likely was mainly due to the aridity and soil infertility of the region [54,56,57]. The modern mean annual rainfall of the study region is only 300–500 mm, and thus it belongs to the arid and semi-arid climate zone (Fig 1). Therefore, drought-tolerant crops, foxtail millet and common millet, which are C_4_ plants with a short maturation time, were more suitable for cultivation than C_3_ crops such as wheat and rice, which need more water for yield maintenance [59–61]. In fact, highly drought-tolerant foxtail and common millet were probably the main crops for the last several thousand years until large-scale cultivation of high-yielding maize was adopted in the agro-pastoral zone in 1960s.

In addition to foxtail and common millet, both wild and cultivated soybean were found at three sites (Xinhua, Zhukaigou, Shimao) (Table 2); however, their size is small compared to modern cultivated varieties [62]. The finding of soybean in the arid agro-pastoral zone, outside the humid monsoon area which is the natural habitat of wild soybean, indicates that the species was domesticated in China since the Yangshao period (Fig 3). *Chenopodium* seeds were found at five sites, and were well represented at Xinhua and Erliban, which may indicate that several species of edible wild *Chenopodium* were used for food and forage in the Neolithic and early Bronze Age in the area. Several drupe pits and nut shell fragments were also found at the sites, including *Prunus armeniaca*, *Prunus persica*, *Castanea* and *Corylus* (Fig 2, Table 2). Among them, apricot was present in the highest proportion, showing that fruit trees occupied an important place in the agricultural system. Like soybean, the natural distribution of these fruit trees is within the humid and semi-humid zones of the monsoon region. Given the evidence for possible forest management and horticultural technology of late Neolithic peoples reported in several studies [54,63], the presence of the various nut shells and drupe pits is strong evidence for early fruit-tree cultivation and management during the Neolithic to Bronze Ages in the agro-pastoral zone of China.

**Fig 6:**
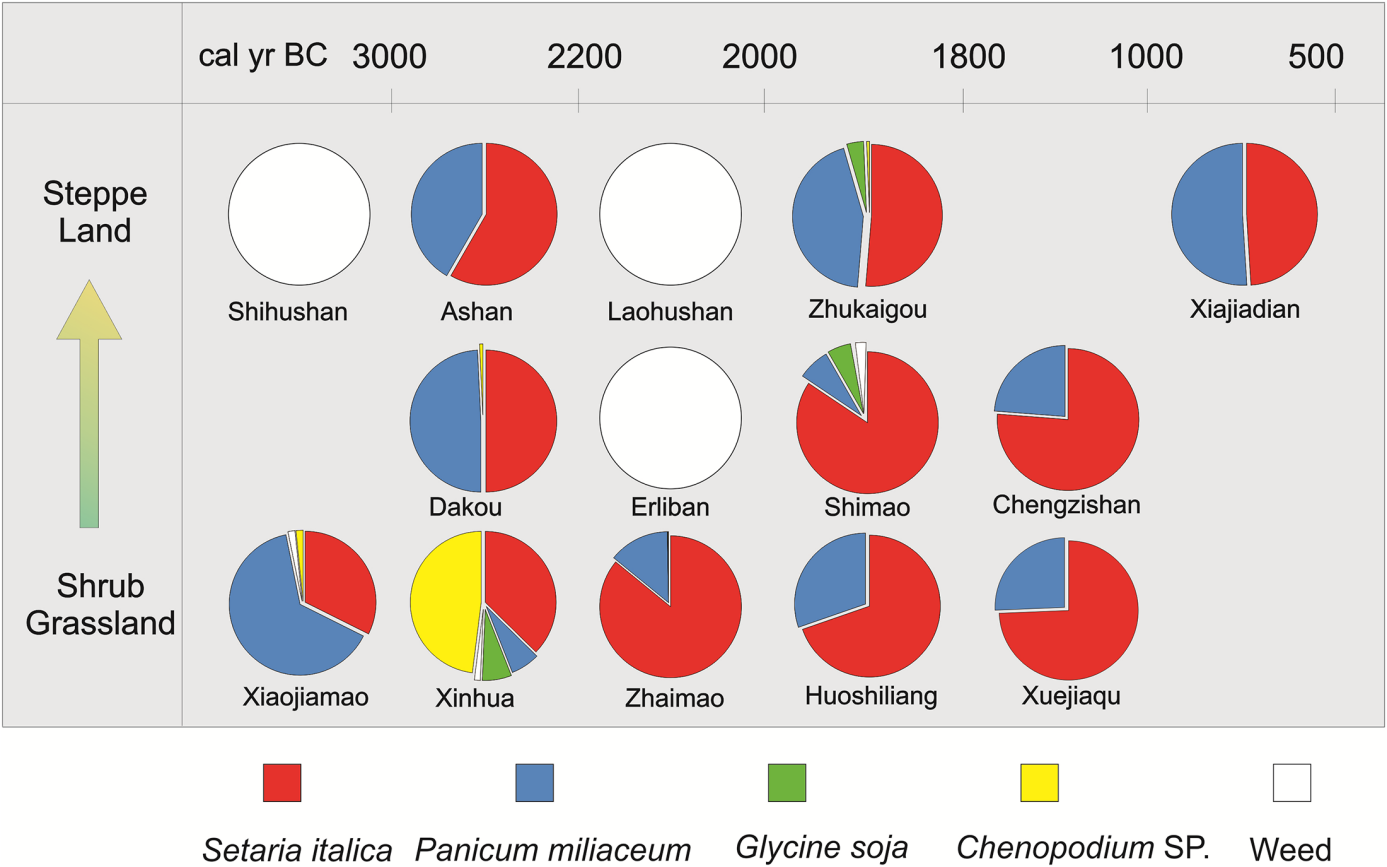
Pie charts showing the composition of assemblages of floated charred seeds from Neolithic to Bronze Age sites in different vegetation zones of the agro-pastoral zone of China. The four taxa are crops and plants characteristic of rain-fed agriculture. From bottom to top, the vegetation zones range from shrub grassland in the northwest, to steppe in the southeast; they represent different monsoon regimes within the study area (see text for explanation).

Our excavation record shows that the studied sites date mainly from the Longshan period to the Western Zhou Dynasty (Fig 2). For example, Shimao is one of the best-known sites and the largest town sites discovered to date in the prehistoric cultural region in the northern China grassland zone[64–66]. The evident prosperity of Shimao during this period shows that settlement of the northern grassland region was developing rapidly and that the population of the sites was increasing substantially. However, as was likely the case for Shimao city, the communities occupying these sites must have experienced significant pressure from the rapid growth in population—with implications for the viability of the overall economy, provision of habitations, and agricultural productivity. Thus, the early agricultural practices at these sites likely reflect an adaptation to population growth (Fig 6). The first adaptation reflects a change in the structure of dryland agriculture, with an emphasis on the cultivation of foxtail millet and common millet.

The relative proportion of foxtail and common millet seeds from the sites varied significantly over time and space. In the early stage, common millet comprised only 30% of the total; subsequently, during 5000 BC-1000 BC, the proportion of foxtail millet increased substantially. Moreover, there were also significant spatial differences (Fig 6). A higher proportion of foxtail millet generally occurred in the southern shrub-grassland area, while there was a higher proportion of common millet in the northern steppe area.

Environmental differences were the likely cause of the differences between the principal crop plants in the northern and southern parts of the agro-pastoral zone. Common millet is easy to cultivate and it appears to have been better suited to primitive agricultural practices than other crops. Under favorable environmental conditions, however, it may be a poor choice because of its limited response to high soil fertility and abundant water availability [59]. In contrast, foxtail millet has a high yield, typically twice that of common millet, and is better adapted to low temperatures. The germination temperature of foxtail millet is 15–25°C, and the jointing temperature is about 22-25°C. By comparison, common millet needs temperatures of 15–20°C to germinate and 25–30°C to joint [40,60,61]. However, foxtail millet requires higher soil fertility and more abundant water than common millet, as well as a longer growing period [40].

The study area experienced an interval of cold and humid climate during 3000 BC-1000 BC [39] and we divided it into three regions reflecting the trajectory of the summer monsoon (Fig 6). The southern part was occupied by shrub grassland with higher precipitation and soil land fertility, while the northern steppe land was drier and less fertile. Thus, foxtail millet must have been selected and cultivated on an increasing scale through the Neolithic and Bronze Ages in the agro-pastoral zone. However, common millet, which is more tolerant of drought and low soil fertility, continued to be important in the northern steppe area. This may indicate that the early farmers were able to adapt continuously to the environment as the human population increased [39].

The second category of evidence for agricultural adaptation to increasing human population is crops yield. There were two aspects to changing crop yield: changes in grain morphology and biomorphic selection [61]. The morphological data show that the size of common millet seeds increased during 3500 BC-2000 BC. The length and width of the seeds of both common millet and foxtail millet were similar at Xiaojiamao site, 3000 BC (Fig 3). However, from 3000–1800 BC, conscious selection probably resulted in the observed increase in the size of common millet seeds which may also have been accompanied by increasing yields [67,68]. From Xiaojiamao to Zhukaigou, the length of common millet seeds increased by ~20%, and the width by ~35%, on average. It can be estimated that both average seed volume and the one-thousand grain weight of common millet in the Longshan period were twice that in the Yangshao period. Together with a possible increase in the average number of grains per common millet plant, we speculate that the average annual common millet yield per unit area may have doubled from 3000 BC-1800 BC in the agro-pastoral zone of China.

It is noteworthy that the percentage representation of foxtail millet increases sharply (Table 3); however, the seed size of foxtail millet is inversely proportional to its representation among the total number of seeds. For example, at Zhaimao (Table 1), very small seeds comprise 86% of the total of foxtail millet seeds, which is the second highest value at all the sites. In contrast to common millet, rice, and sorghum, foxtail millet cultivars can produce 1–3 spike ears, so that the number of grains per plant has a wide range of variability [40]. Therefore, there is a significant possibility that although the yield of foxtail millet may also have increased within a similar range to common millet, the increase in the number of seeds per plant may be the main reason for the increase. Moreover, this may be the cause of the increase in the proportion of foxtail millet within the total seed number of seeds.

## Conclusions

Based on an analysis of fossil seeds at archaeological sites, we have studied changes in the proportions of crops cultivated in the arid and semi-arid transition zone of northern China during 5000 BC-1000 BC, spanning the Yangshao period to the Western Zhou dynasty. Due to the regional environment, climate and soil fertility, wheat and rice were not the main crops, and the typical rain-fed cereals, foxtail millet and common millet, occupied the dominant position in the agricultural system.

The proportion of foxtail millet increased substantially from the Yangshao to the Longshan period. In the southern shrub-grassland region of the agro-pastoral zone, foxtail millet, rather than common millet, became the primary food crop in the late Longshan period. In contrast, in the arid northern steppe region, common millet remained important in the Longshan and Bronze Age. Soybean played an important role in the dryland agricultural system, probably representing the earliest cultivated soybean species. Fruit trees, such as apricot, peach, and chestnut, may have been cultivated at the same time. These characteristics may reflect an adaption of the agricultural system to the increasing human population size during 3000 BC-1000 BC.

Analysis of the changes in seed morphology indicates an increase in the size of the seeds of common millet, especially, which may reflect an adaptation to the rapidly increasing human population from 3000 BC-2000 BC. These changes likely resulted in a significant improvement in the dryland agricultural productivity in the agro-pastoral zone. If we assume that the cultivated area was relatively constant, the average yield per unit area in the late Longshan may have been twice that of earlier periods. In addition, the increase in the productivity of millet cultivation may have promoted its spread from north China westwards to Eurasia with the result that it became a global crop by around 2000 BC.

## Supporting information

S1 File Lab calibration data (original). This is the raw data we obtained from ANSTO and BETA laboratory, including a variety of measurement results. And in this paper, we only selected the ^14^C dating part.
(XLSX)

S2 File Lab calibration data (after arrangement). (XLSX)

S3 File Seed morphology data. (XLSX)

S4 File ^14^C calibration plots of samples. (RAR)

S5 File Seed identification under stereoscope. (RAR)
